# Measurement Invariance of the Questionnaire on the Internal Stigma of Internet Surfing Among Sino-Australian Undergraduates

**DOI:** 10.3389/fpsyt.2022.823504

**Published:** 2022-02-08

**Authors:** Jie Luo, Ying Ge, Ji-chun Hao, Ross B. Wilkinson, Jay L. Wenger

**Affiliations:** ^1^School of Psychology, Guizhou Normal University, Guiyang, China; ^2^Key Laboratory of Emotion and Mental Health in Chongqing, Chongqing University of Arts and Sciences, Chongqing, China; ^3^School of Psychology, The University of Newcastle, Newcastle, NSW, Australia; ^4^School of Humanities and Social Sciences, Fuzhou University, Fuzhou, China; ^5^Social Sciences Division, HACC, Central Pennsylvania's Community College, Lancaster, PA, United States

**Keywords:** stigma, internal stigma of Internet surfing, QISIS, measurement invariance, Sino-Australian undergraduates

## Abstract

**Background:**

The stigma of internet surfing is a relatively new area of study arising from the popularity of the internet. The Questionnaire on the Internal Stigma of Internet Surfing-9 (QISIS-9) was developed for the Chinese culture, so its suitability for use in other cultural contexts is uncertain. This paper examines the measurement invariance of the QISIS-9 among Sino-Australian undergraduates to verify the cross-cultural measurement invariance of QISIS-9 and promote cross-cultural (nationality) research regarding the internal stigma of internet surfing.

**Methods:**

The Internal Stigma of Internet Surfing-9 (QISIS-9) was used to assess 200 Chinese undergraduates (50% female, M_age_ = 19.78) and 204 Australian undergraduates (76% female, M_age_ = 21.10), respectively.

**Results:**

A confirmatory factor analysis (CFA) indicated that the single-factor model of QISIS-9 is acceptable with both Chinese and Australian undergraduates. However, the factor loading of Item 9, to which a reverse score is assigned, is not ideal for both samples. Thus, the item should be deleted. According to a multigroup confirmatory factor analysis (MCFA), QISIS-8, the revised version of QISIS-9, meets the strict measurement invariance among the Chinese and Australian participants. The QISIS-8 demonstrated appropriate internal consistency in the scores for both the Chinese and Australian undergraduates.

**Conclusion:**

The new QISIS-8 can effectively assess the internal stigma of internet surfing among Chinese and Australian undergraduates, and it provides a frame of reference for further cross-cultural (border) comparisons.

## Introduction

Thirty-five percent of the world's population uses the internet, half of whom are youth. The latest internet report, produced in 2021, shows that 33.0% of netizens are under age 29 (1). According to a 2016 survey, 83% of Australian youth surfed the internet about three times a day and spent an average of 14 h each month on the internet (2). This amount of internet use contributes enormously to the study and life of undergraduates. However, excessive use of the internet has its negative effects, such as internet addiction, social alienation and stigma of internet surfing etc. that associate internet surfing with mental health problems. Negativity and facilitation coexist in the development of the internet. Identifying the patterns of use and guiding netizens into using the internet in positive ways are worthwhile pursuits.

China and Australia are home to a slew of data centers, but they have different cultures and eco-environments. Chinese culture is a collectivistic cultural model based on Han culture, and cognitive modes tend to converge and conform. Australia is a country of immigrants, where people of all races live together, but the western individualistic cultural system is dominant, and cognitive judgments and value evaluations are more personalized (3). Therefore, it is of great theoretical and practical significance to study the status quo and characteristics of the internal stigma of internet surfing among Sino-Australian undergraduates from a cross-cultural perspective.

### Stigma

Goffman (4) was the first to frame the concept of stigma. He defines it as the derogatory and humiliating label that society attaches to individuals or groups who display conditions, attributes, quality, characteristics, or behaviors unacceptable in their own culture. Early research on stigma focused on individuals' unacceptable characteristics (4). Later, some scholars (5, 6) revised the concept and pointed out that this attribute conveys a depreciated social identity. The concept of stigma was first introduced to the Chinese academic community by Xie (7). Around 2000, the Chinese Mainland began research on stigma in medical science, sociology, social psychology, and anthropology. Guan (8) and Guo (9), for example, studied the conceptual characteristics of stigma and explored ways of stigma model building (8, 9). Stigma is generally regarded as a multidimensional composite comprising stereotype (negative cognitive appraisal), prejudice (negative emotional response), and discrimination (disposition to discriminating behavior) (5, 10).

The stigmas of physical and mental illnesses are foremost in Chinese and international research on stigmas (10–16). Research on the stigma of social identity comes in second place, covering race / nationality, sex, and special groups. The stigma of race/nationality has long been a hot topic among Western scholars (17–19). Western and Taiwanese researchers often study the stigma of gender discrimination from the feminist perspective (20–22), and most scholars on Chinese Mainland study the stigma of sex from the perspective of gender stereotypes (23–26). Western scholars look closely at homosexuals and drug abusers in their research of special groups (27–31), while Chinese scholars pay more attention on local issues, such as migrant workers (32–35), migrant populations (36, 37), and phoenix men (38–40). A new term, the stigma of internet surfing, has been coined amidst the rapid development of the internet and its potentially negative effects.

### Stigma of Internet Surfing

The stigma of internet surfing is a relatively new area of study arising from the popularity of the internet. It was coined by Chinese scholar Lei et al. (41) to describe the negative stereotypes, prejudices, and discrimination of various internet behaviors on home computers and mobile devices (41). Lei and his partners (2012) developed the Questionnaire on the Internal Stigma of Internet Surfing among teenagers (QISIS) – adapted from a prior scale (e.g., internalized stigma of mental illness, ISMI) (42) and evaluated with the teenagers. The original QISIS was a four-point Likert-type scale including 13-item that loaded on a single factor: internal stigma of internet surfing (ISIS). The subsequent QISIS that consisted of 9 items, was developed by means of the exploratory and confirmatory factor analyses. The CFA indices for the unidimensionality of the QISIS were adequate (e.g., RMSEA = 0.051, CFI = 0.950, TLI = 0.936), and the internal consistency coefficients of the QISIS were acceptable to good (e.g., split-half reliability was 0.748, and the alpha coefficient was 0.855). Results indicated that girls and senior students display a higher degree of cognition of the stigma of internet surfing. Apparently, internet addiction is a good predictor of the stigma of internet surfing and highly addicted individuals show a higher degree of internal stigma (43). Lei and colleagues also found that the cognition of stigma provides the basis and conditions for the occurrence of the internal stigma of internet surfing. The internal stigma of internet surfing refers to the negative self-appraisal, self-perception and behavioral tendencies that individuals form after internalizing society's negative comments on and cognition of their internet behaviors (41).

Lei et al.'s (41) research shows that teenagers' cognition of the stigma of internet surfing and their internal stigma increase with age. Their developing self-awareness may lead to an increased comparison of the difference between their group and other groups as their cognition abilities improves and knowledge grows (41). In addition, Ugrin and Pearson (44) have been exploring ways to minimize overuse of the internet from the perspective of organizational management (44). Peter et al. (45) found that people often stigmatize online games because such games can encourage anger and guilt which might lead to serious communication disorders (45).

So far, there has been very little cross-cultural research on the stigma of internet surfing. Therefore, the current study is designed to investigate the stigma of internet surfing, especially the internal stigma of internet surfing, in the context of an oriental culture represented by China and a Western culture represented by Australia. No direct Australian research on the stigma of internet surfing is available, so we will carry out a comparative study of the use of the internet among Chinese and Australian late teenagers.

### Comparison of the Use of the Internet Among Sino-Australian Teenagers and Their Mental Health

About 16.6% of Chinese netizens are under the age of 19, and 17.8% of them are between 20 and 29 (1). Chinese teenagers encounter the internet at a very young age, and the frequency of internet surfing among them increases yearly. The majority access the internet via mobile devices (46). They like information and apps on entertainment, and 62.5% of them play online games (47, 48). A recent survey shows that young students lack enthusiasm for online autonomous learning. Instead, they prefer to entertain themselves when online–specifically online games and interactions on social media dominate their use of the internet (49). Unfortunately, an individual's fear of negative evaluation exacerbates social anxiety, weakens self-control, and can cause excessive internet use (50). In turn, excessive use is often linked with poor academic performance in schools, and Cui et al. (51) found that internet addiction is rampant in less-democratic families (51).

Likewise, the internet is widely accessible in Australia, and 92% of teenagers have access to online education (52). The internet is a young Australian's major means for acquiring knowledge about politics, and it paves the way for young voters to get involved in political campaigns (2). However, excessive access of the internet can have an adverse impact on a teenager's academic performance (53). An extensive study shows that 55.2% of Australian teenagers communicate and play games on the internet, and 58.9% of them are addicted to the internet (54). Australian teenagers who overuse the internet and indulge in online games are more emotionally troubled and prone to problematic behaviors (55, 56). Unlike China, research and intervention programs aimed at addressing internet deviations and problems in Australia are mainly organized and financed by non-profit and private organizations. The government rarely gets involved in these efforts (57).

Young people in China and Australia display different mental health statuses in their cultures. Overall, Chinese and Australian undergraduates show moderate subjective senses of well-being. However, Chinese male students and liberal arts students show a lower subjective sense of well-being. But Chinese female students and science students have a higher subjective sense of well-being than Australian students. Research on affinity, coping strategies and mental stress among Chinese and Australian undergraduates shows that a clingy Australian couple will experience greater mental stress if they retreat and accuse themselves when coping with their problems, while a Chinese couple will experience greater mental stress and confusion only when they adopt the self-accusation strategy (58). According to Hu and Wang (59), Chinese youth who immigrated to Australia over the previous 5 years, often experience considerable mental stress and confusion (59).

### Measurement Invariance

A critical, methodological issue in conducting cross-cultural research is measurement invariance. Measurement invariance describes the consistency in the outcome of tests that involve the same measurement tool and construct across different scenarios or samples such as genders, evaluators, testing media, cultural environments, and groups (60–62). Establishing measurement invariance is crucial for confirming that a measure is consistent across groups (e.g., male vs. female) (63). It is also a criterion for investigating whether a measure has the same functions across groups. In particular, a cross-cultural study should find out if different cultural groups have the same understanding of the same construct (62–64). If a tool that does not meet the requirements for measurement invariance is employed, researchers will be unable to determine whether the differences are trustworthy in their comparison of samples. In our investigation, if the QISIS test does not meet the requirements for cross-sample measurement invariance, we must proceed cautiously when trying to explain cross-cultural differences. If, however, the test meets the requirements for measurement invariance, our results will be more reliable.

The analysis of measurement invariance is currently implemented in two ways (65, 66): (1) multigroup confirmatory factor analysis (MCFA) under the framework of the structural equation model; (2) differential item functioning (DIF) under the framework of the item response theory. The current study adopts MCFA in its analysis of measurement invariance. The MCFA-based test method usually examines the comparison of nested models to establish measurement invariance, which includes configural invariance, weak invariance, strong invariance, and strict invariance. More specifically, configural invariance mainly verifies whether the formation or model of latent variables is the same (factor model equivalency). Weak invariance examines the relationship between observed variables (i.e., items) and latent variables (i.e., factors) based on configural invariance. In other words, it tests whether factor loading is invariant across groups. Strong invariance checks whether the intercept of observed variables (items) is invariant across groups based on weak invariance. Strict invariance further analyzes whether the error variance of observed variables (items) is invariant across groups based on strong invariance. Strict invariance suggests that the difference in scale score variation across groups reflects the difference in latent variable variation across groups.

As stated, the tool to test the internal stigma of internet surfing was first developed by Lei et al. (41) in their research with adolescents (41). Since it remains unknown to what degree the tool is applicable to undergraduates and what its psychometric properties are among Australian subjects, it cannot be applied in cross-cultural studies. To make the comparison of the internal stigma of internet surfing among Sino-Australian undergraduates applicable and effective, we will examine the measurement invariance of QISIS-9 among Sino-Australian undergraduates with a view to laying psychometric foundations for follow-up comparisons of the internal stigma of internet surfing among Chinese and Australian undergraduates.

## Methods

### Participants

#### Chinese Samples

An offline survey was conducted among undergraduates at Chongqing University of Arts and Sciences in China. A total of 200 participants (50% female; 18–23 years old; mean age = 19.78; SD = 1.30) were recruited to complete the questionnaires, including 100 liberal arts students and 100 science students; 50 were freshmen, 66 were sophomores, 69 were juniors, and 15 were seniors. Additionally, several of participants were randomly selected for follow-up interviews based on the scores of the QISIS.

#### Australian Samples

An online survey was conducted among undergraduates at the University of Newcastle in Australia. A total of 204 participants (76% female; 18–30 years old; mean age = 21.10; *SD* = 3.28) were returned. In the current sample, 192 were from Europe and North America, 10 were from Asia, and 2 didn't indicate their nationality. All were freshmen. Moreover, several of participants were randomly selected for follow-up interviews based on the scores of the QISIS.

### Procedure

The appropriate IRB approval was sought and obtained from the Chongqing University of Arts and Sciences (Review NO. 20180614) and the University of Newcastle (Reference NO. H-2018-0293), respectively. All Chinese participants provided written consent prior to completing the questionnaire, and all the Australian participants provided online informed consent prior to participation. Participants were informed that the study was voluntary, and they could discontinue at any time. In addition, participants were informed that their answers would remain anonymous and were invited to ask questions regarding the investigation.

### Measures

The Questionnaire on the Internal Stigma of Internet Surfing-9 (QISIS-9) was developed by Lei et al. (41). It is a unidimensional questionnaire that covers nine items. Each item is scored on a four-point Likert-type scale from 1(*completely disagree*) to 4 (*completely agree*). The English version of the QISIS-9 was translated into English by the first and second authors, and was back translated into Chinese by the second and third authors. The final version was jointly produced by a Chinese psychology professor (the second author) and an Australian psychology professor (the fourth author) through inter-translation.

### Statistical Analysis

The SPSS 22.0 was used to input the survey data, perform the descriptive statistics, and examine the internal consistency coefficient. The Mplus 8.0 was used to carry out the confirmatory factor analysis (CFA) and measurement invariance analysis. The following steps were taken in the analysis:

First, we performed the descriptive statistics of QISIS-9 that Chinese and Australian undergraduates get on the mean, standard deviation, skewness and kurtosis (see Table 1). Because the scores of the Chinese samples were beyond the range of −1 to +1 on skewness and kurtosis for all QISIS-9 items (except for Item 9), the MLM (maximum likelihood estimation with a mean-adjusted chi-square), was adopted in the confirmatory factor analysis (CFA) to examine the factor structure of the QISIS-9. It is robust to non-normality. The CFA was performed based on the following indicators (67): comparative fit index (CFI; ≥0.90 suggests an acceptable model fit), Tucker-Lewis index (TLI; ≥0.90 suggests an acceptable model fit), root-mean-square error of approximation (RMSEA; ≤ 0.08 indicates an acceptable model fit), and standardized root mean square residual (SRMR ≤ 0.08 indicates an acceptable model fit).

**Table 1:** Descriptive statistics of scores of Sino-Australian undergraduates in QISIS-9.

**Item**	**China**							**Australia**						
	***Min***	***Max***	***M***	***SD***	***SK***	***KU***	**CITC**	***Min***	***Max***	***M***	***SD***	***SK***	***KU***	**CITC**
Item-1	1	4	1.47	0.72	1.60	2.41	0.71	1	4	2.15	0.74	0.33	−0.05	0.67
Item-2	1	4	1.56	0.77	1.14	0.35	0.72	1	4	2.21	0.73	0.34	0.06	0.74
Item-3	1	4	1.48	0.67	1.40	1.84	0.72	1	4	2.21	0.70	0.03	−0.36	0.72
Item-4	1	4	1.51	0.73	1.47	1.89	0.70	1	4	2.11	0.78	0.37	−0.17	0.67
Item-5	1	4	1.55	0.74	1.33	1.43	0.65	1	4	2.07	0.75	0.25	−0.34	0.64
Item-6	1	4	1.52	0.76	1.41	1.34	0.59	1	4	1.98	0.67	0.32	0.16	0.65
Item-7	1	4	1.28	0.58	2.43	6.78	0.73	1	4	2.01	0.73	0.29	−0.24	0.72
Item-8	1	4	1.37	0.61	1.57	1.96	0.72	1	4	1.88	0.69	0.62	0.82	0.74
Item-9	1	4	2.24	0.92	0.17	−0.88	0.17	1	4	2.33	0.69	−0.18	−0.44	−0.13

*SK, kewness; KU, kurtosis; CITC, Corrected Item Total Correlations*.

Second, the measurement invariance (MI) of QISIS-9 between Chinese and Australian samples was examined. In other words, multigroup confirmatory factor analysis (MCFA) was carried out successively to check the configural invariance, the weak invariance, the strong invariance and the strict invariance. Since the Chi-square difference test is susceptible to sample size (68), the present research will examine difference scores in CFI (ΔCFI), TLI (ΔTLI), and RMSEA (ΔRMSEA) to evaluate MI. According to Cheung and Rensvold (69), ΔCFI and ΔTLI <0.01 supports MI (69). Chen (68) suggests ΔRMSEA <0.015 supports MI (68). Following the MI, the latent means among Sino-Australian undergraduates were calculated to compare the differences between Chinese and Australian undergraduates. Specifically, the latent mean scores were computed by setting the Chinese group as the reference group (i.e., setting the QISIS mean to zero in the Chinese group) and freely estimating the latent means in the Australian group.

Third, the alpha coefficient and mean inter-item correlations (MIC) were used to examine the internal consistency of the QISIS-9 scores. An alpha coefficient bigger than 0.70 is acceptable (70), and a value of MIC within the range from 0.15 to 0.50 suggests satisfactory internal consistency (71).

## Results

### Descriptive Statistics

Means, standard deviations, skewness, kurtosis, and correlations between items and total scale scores are shown in Table 1. The scores of Chinese participants on all QISIS-9 items show a positive skewed distribution (except for Item 9), indicating that the Chinese undergraduates have a relatively low score on the internal stigma of internet surfing and the scores that Australian undergraduates get on all QISIS-9 items show a normal distribution.

### Factor Structure of the QISIS-9 in Sino-Australian Sample

The CFA indicated two findings: (1) the QISIS-9 shows unidimensionality among the Chinese undergraduates, all the fit indexes are acceptable (χ^2^ = 84.366, *df* = 27, RMSEA = 0.063, CFI = 0.958, TLI = 0.945, SRMR = 0.041), and the factor loading value of all items is ideal (except for Item 9, the factor loading value of which is 0.171) (see Table 2); (2) QISIS-9 also shows unidimensionality among the Australian undergraduates (χ^2^ = 129.766, *df* = 27, RMSEA = 0.109, CFI = 0.909, TLI = 0.878, SRMR = 0.054), and the factor loading value of all items is satisfactory (except for Item 9, the factor loading value of which is −0.11) (see Table 2). These results indicate that the factor loading of Item 9 among the Chinese and Australian undergraduates does not reach an acceptable level (<0.30). Thus, another CFA was performed excluding Item 9. The results indicate that: (1) the QISIS-8 shows unidimensionality among the Chinese undergraduates (χ^2^ = 76.552, *df* = 20, RMSEA = 0.067, CFI = 0.960, TLI = 0.944, SRMR = 0.041), and the factor loading of all items is ideal (see Table 2); (2) the QISIS-8 shows an acceptable unidimensionality among the Australian undergraduates (χ^2^ = 113.734, *df* = 20, RMSEA = 0.116, CFI = 0.920, TLI = 0.888, SRMR = 0.052), and the factor loading of all items is satisfactory (see Table 2). Therefore, the QISIS-8 would be used in subsequent analysis of the measurement invariance.

**Table 2:** Factor loading of items in QISIS-9 and QISIS-8.

**Item**	**China**		**Australia**	
	**QISIS-9**	**QISIS-8**	**QISIS-9**	**QISIS-8**
Item 1: I think I am useless when spending time on the Internet	0.56	0.56	0.56	0.56
Item-2: I think less of my abilities when spending time on the Internet	0.61	0.61	0.59	0.59
Item-3: I feel negative about myself when spending time on the Internet	0.52	0.52	0.58	0.57
Item-4: I feel inferior to others when spending time on the Internet	0.56	0.56	0.57	0.57
Item-5: I lower my self-expectation when spending time on the Internet	0.52	0.52	0.48	0.48
Item-6: I feel upset when spending time on the Internet.	0.48	0.48	0.46	0.46
Item-7: I feel guilty or embarrassed when spending time on the Internet	0.44	0.44	0.54	0.54
Item-8: I feel worthless when spending time on the Internet	0.46	0.46	0.52	0.52
Item-9: I feel confident about myself when spending time on the internet	0.17		−0.11	

*QISIS, Questionnaire of the Internal Stigma of Internet Surfing*.

### Measurement Invariance

Taking previous methods in cross-cultural studies of measurement invariance (72, 73) as reference, the present study adopts the MCFA to test the MI of the scores that the Chinese and Australian undergraduates get in QISIS-8 (see Table 3).

**Table 3:** Measurement invariance model fit statistics for the QISIS-8 across Chinese and Australian undergraduates.

**Model**	**χ^2^**	***df***	**CFI**	**ΔCFI**	**TLI**	**ΔTLI**	**RMSEA**	**ΔRMSEA**
Configural invariance	190.287	40	0.938	-	0.914	-	0.092	-
Weak invariance	197.720	47	0.936	−0.002	0.924	0.010	0.086	−0.006
Strong invariance	219.316	54	0.925	−0.011	0.923	−0.001	0.087	0.001
Strict invariance	245.133	62	0.925	0.000	0.932	0.009	0.081	−0.006

*Df, degrees of freedom; CFI, comparative fit index; TLI, Tucker–Lewis index; RMSEA, root mean square error of approximation; ΔCFI, change in comparative fit index relative to the preceding model; ΔTLI, change in Tucker-Lewis index relative to the preceding model; ΔRMSEA, change in root mean square error of approximation relative to the preceding model*.

#### Configural Invariance

Factor loading and intercept are roughly estimated and no assumption about identity is made in cross-cultural samples in investigating the configural invariance (test whether the form or model of latent variables is the same), so as to establish a baseline model for subsequent comparison of nested models. Table 3 suggests that all the fit indexes of configural invariance in the present study basically meet psychometric requirements (e.g., CFI and TLI > 0.90) and that the scores of the Chinese and Australian undergraduates in QISIS-8 meet the requirements for configural invariance.

#### Weak Invariance

The same factor loading is set for the two sample groups to test the weak invariance after configural invariance was established. Table 3 indicates that all the fit indexes of weak invariance meet psychometric requirements (e.g., CFI and TLI > 0.90). Although the value of ΔTLI is 0.01, the value of ΔCFI is smaller than 0.01, and the value of ΔRMSEA is smaller than 0.015. This pattern of results suggests that the scores of the Chinese and Australian undergraduates on the QISIS-8 meet the requirements for weak invariance.

#### Strong Invariance

The same measure intercepts are set for the two sample groups to test the strong invariance after weak invariance was established. Table 3 shows that all the fit indexes of strong invariance meet psychometric requirements (e.g., CFI and TLI > 0.90). The value of ΔCFI is −0.011, while the value of ΔTLI is smaller than 0.01 and the value of ΔRMSEA is smaller than 0.015, respectively. This pattern of results suggests that the scores of the Chinese and Australian undergraduates on the QISIS-8 meet the requirements for strong invariance.

#### Strict Invariance

The same measure intercepts are set for the two sample groups to test the strict invariance after strong invariance was established. Table 3 indicates that all the fit indexes of strict invariance in the present study meet psychometric requirements (e.g., CFI and TLI > 0.90), the value of ΔCFI and ΔTLI is smaller than 0.01, and the value of ΔRMSEA is smaller than 0.015. This pattern of results reveals that the scores of the Chinese and Australian undergraduates on the QISIS-8 meet the requirements for strict invariance.

When the strict invariance was established, the differences in the scores that Chinese and Australian undergraduates get in QISIS-8 were examined. The results show that the Australian undergraduates manifest significantly higher scores in QISIS-8 than Chinese undergraduates do (mean difference = 0.669, *p* < 0.001).

### Internal Consistency of QISIS

The alpha coefficient and the MIC were adopted to examine the internal consistency of QISIS-9 among the Chinese and Australian undergraduates. We have two findings: (1) The alpha coefficients of the scores that the Chinese and Australian undergraduates get for the QISIS-9 are 0.87 and 0.86, and the MICs are 0.46 and 0.41, both respectively; (2) After Item 9 was deleted in view of the results of the CFA, the alpha coefficients of the scores of the Chinese and Australian undergraduates in QISIS-8 are 0.91 and 0.91, and the MICs are 0.56 and 0.56, both respectively.

## Discussions

The QISIS-9 was developed for the Chinese culture, so its suitability for use in other cultural contexts is uncertain. The present study is the first to take samples from Chinese and Australian undergraduates in an effort to verify the cross-cultural measurement invariance of QISIS-9 and promote cross-cultural (nationality) research regarding the internal stigma of internet surfing. The results show that the QISIS-8, a revised version of QISIS-9 where Item 9 is deleted, is reliable and valid among both Chinese and Australian undergraduates. Furthermore, the revised QISIS-8 meets the strict measurement invariance across Sino-Australian undergraduates and demonstrates satisfactory internal consistency in the two groups.

Given that the QISIS-9 was originally based on Chinese senior high school students (41), its psychometric properties among undergraduates needed to be verified. Accordingly, the reliability and validity of the QISIS-9 among Chinese and Australian undergraduates were examined before the cross-cultural invariance analysis was conducted. The CFA and internal consistency coefficients demonstrate that the psychometric properties of QISIS-9 among the Chinese and Australian undergraduates are comparable and acceptable. But interestingly, the reverse-coded item (Item 9, “I feel confident about myself when spending time on the internet”) in QISIS-9 was inferior to the other eight items (positively-coded for the internal stigma of internet surfing). More specifically, the reliability and validity, especially the value of the alpha coefficient, of QISIS-8 improved in CFA and internal consistency after deleting the reverse-coded Item 9. There are disagreements about whether reverse scored items should be used in questionnaires. For example, some scholars (74, 75) argue that such items may have a negative impact on the psychometric properties of questionnaires. Likewise, others (76, 77) point out that reverse scored items may have an impact on the factor structure of measurement tools (e.g., item wording effect). Following those studies, the current study also found that Item 9, a reverse-coded item in QISIS-9, had a negative impact on the psychometric properties of the QISIS-9. Thus, Item 9 was deleted, and the revised QISIS-8, which covers eight items, was developed and used to analyze the measurement invariance among the Sino-Australian undergraduates. This development will promote cross-cultural (nationality) research and comparison of the internal stigma of internet surfing.

The MCFA indicated that the strict measurement invariance (specifically configural, metric, scalar, and strict invariance) of the QISIS-8 across Chinese and Australian undergraduates has been observed. More specifically, the configural invariance suggests that the QISIS-8 has the same unidimensional structure across Chinese and Australian samples; the metric invariance demonstrates that the QISIS-8 has the same factor loadings among Chinese and Australian populations; the scalar and strict invariance further supports that the QISIS-8 has the same intercepts and residual invariance across Chinese and Australian undergraduates, respectively. Overall, the QISIS-8 supported the measurement invariance among the Chinese and Australian participants, suggesting that the QISIS-8 is simultaneously applicable to the investigation and assessment of internal stigma of internet surfing among the Chinese and Australian undergraduates. Thus, the mean difference in internal stigma of internet surfing scores as measured by the QISIS-8 can be interpreted as the true difference in the level of internal stigma of internet surfing between Chinese and Australian undergraduates.

Based on the results of the strict measurement invariance, we subsequently compared the scores of Chinese and Australian undergraduates on internal stigma of internet surfing and found that the QISIS-8 scores of the Australian undergraduates were significantly higher than those of the Chinese students. This is evidence that internal stigma of internet surfing likely exists among the Australian undergraduates, and is different than China's situation. For stigmatization, Chinese undergraduates tend to internalize their stigma of internet surfing with a perspective of conformity and a general “public stigma.” Public stigma is a continuous process in which the public stigmatizes some specific groups, events or behaviors (9). “Collective-centered” individuals accept and normalize public stigma based on collective identity thinking (78). Australian undergraduates, however, stigmatize internally the internet surfing when they are unable to cope with the physical and mental damage caused by improper internet use, and this is magnified by an individualistic culture – one that emphasizes individual responsibility and selection. This stigmatization process is based on individual self-recognition and judgment after online practice and experience (79).

A recent study has pointed out that 80% of Australian youth play games online and gamble, and 1–5% of them are addicted to these kinds of activities (80). These Australian youth enjoy the convenience and excitement that the internet brings, but they are not well-equipped to deal with the psychological problems that arise therein. They do not have effective coping strategies.

In a developed country of immigrants, the youth of multi-cultural Australia have a significant prevalence of psychological distress and tendencies toward stigmatization (81). Youth with mental disorders are reluctant to seek help, and they lack relevant knowledge to adequately deal with their own problems (82–84). We also found from interviews with participants that while internet surfing can bring pleasure; it can also produce self-accusations and anxiety. Mental frustrations have emerged, leading many youths to believe that the internet is wasting their time, money, and energy. And they have no idea how to use the internet scientifically and reduce its harm to their physical and mental health. This gives rise to the internal stigma of internet surfing. Australian youth definitely need to use information technology and the internet in more positive ways – green, environmentally-friendly, and sustainable ways. Hopefully, corresponding mental problems can be addressed (85).

### Limitations and Future Directions

The findings of this study should be considered in light of its limitations. First, participants were predominantly recruited from the southwest of China and New South Wales in Australia. Thus, the results might not generalize to other geographic areas or cultures. Additional studies should further examine and replicate our findings in other regions in China and other Western samples (e.g., North America). Moreover, the QISIS was originally developed based on Chinese senior high school students. Future research should benefit from investigating cross-cultural invariance of the QISIS by comparing a sample of Chinese youth directly with Western samples. Finally, data collection (offline / online) and sample characteristics (e.g., sex ration, and age) were different in both groups (e.g., Chinese and Australian populations), and these differences might influence the results of invariance across the groups. Future studies should be focus on the aforementioned factors to avoid their influence on the measurement invariance and comparison of differences in cross-cultural research.

Despite these limitations, results of the current study have suggested that the revised QISIS-8 demonstrates strict measurement invariance across Sino-Australian undergraduates, as well as satisfactory internal consistency. It holds promise as a self-report instrument for the assessment of internal stigma of internet surfing among Chinese and Australian undergraduates.

## Data Availability Statement

The data supporting the conclusions of this study are available upon request to author YG (gy8620@163.com) and corresponding author RW.

## Ethics Statement

The studies involving human participants were reviewed and approved by the Chongqing University of Arts and Sciences Institutional Review Board. The University of Newcastle Human research Ethics Committee. The patients/participants provided their written informed consent to participate in this study.

## Author Contributions

YG was mainly responsible for the overall conception and design of this study, investigated the data, drafted the manuscript, and provided the final approval for the manuscript. JL contributed to the analysis and interpretation of the data, drafted the manuscript and provided the final approval for the manuscript. J-cH helped to carry out the investigation of the data. RW contributed to the investigation of the data, and revised the manuscript. JW also revised the manuscript and provided final approval for the manuscript. All authors contributed to the article and approved the submitted version.

## Funding

This study was funded by the China National Scholarship Foundation (201708505119).

## Conflict of Interest

The authors declare that the research was conducted in the absence of any commercial or financial relationships that could be construed as a potential conflict of interest.

## Publisher's Note

All claims expressed in this article are solely those of the authors and do not necessarily represent those of their affiliated organizations, or those of the publisher, the editors and the reviewers. Any product that may be evaluated in this article, or claim that may be made by its manufacturer, is not guaranteed or endorsed by the publisher.

## References

[1] China Internet Network Information Center, CNNIC. The 48th China Statistical Report on Internet Development [OB/OL]. (2021). Available online at: Available online at: http://www.cnnic.net.cn/hlwfzyj/hlwxzbg/hlwtjbg/202109/P020210915523670981527.pdf (accessed September 15, 2021).

[2] McAllister T. Internet use, political knowledge and youth electoral participation in Australia. J Youth Stud. (2016) 19:1220–36. 10.1080/13676261.2016.1154936

[3] Chiu C Y Hong YY. Social psychology of culture. London: Psychology Press (2006).

[4] Goffman E. Stigma: Notes on the Management of Spoiled Identity. Englewood Cliffs, NJ: Prentice Hall, Inc., Englewood Cliffs (1963).

[5] Corrigan PW. How stigma interferes with mental health care. Am Psychol. (2004) 59:614–25. 10.1037/0003-066X.59.7.614 15491256

[6] Crocker J Major B Steele C. Social stigma. In: Gilbert DT, Fiske ST, Lindzey G. The handbook of social psychology. New York, NY: McGraw-Hill (1998).

[7] Xie S. Ethnic Contacts, Stigmatized Identity, and Pan-Taiwan Aboriginalism: A Study on Ethnic Change of Taiwan Aborigines. Taipei: Independence Evening Post (1987).

[8] Guan J. Evolution of stigma as a concept and construction of a multidimensional model. J Nankai Univ. (2007) 14:126–34. 10.3969/j.issn.1001-4667.2007.05.017

[9] Guo JH. Study of stigma: evolution of concept, theories, and model. Academia Bimestrie. (2015) 26:99–109. 10.3969/j.issn.1001-9790.2015.02.011

[10] Corrigan PW Markowitz FE Watson AC. Structural levels of mental illness stigma and discrimination. Schizophr Bull. (2004) 30:481–91. 10.1093/oxfordjournals.schbul.a007096 15631241

[11] Cheng Y Yang S Sang Q. Relieving implicit mental illness stigma against college students: A research based on stereotypic explanatory bias. Chin J Spec Edu. (2015) 22:89–96.

[12] Crandall CS Moriarty D. Physical illness stigma and social rejection. Br J Soc Psychol. (2011) 34:67–83. 10.1111/j.2044-8309.1995.tb01049.x 7735733

[13] Fatimah JB Nancy E. Stigma and intersectionality: a systematic review of systematic reviews across HIV/AIDS, mental illness, and physical disability. BMC Public Health. (2018) 18:919. 10.1186/s12889-018-5861-3 30049270 PMC6062983

[14] Liu LX. Research on psychological factors of discrimination against AIDS and people living with HIV. Chin J Public Health Manag. (2020) 36:489–91.

[15] Schomerus G Schwahn C Holzinger A Corrigan PW Grabe HJ Carta MG. Evolution of public attitudes about mental illness: a systematic review and meta-analysis. Acta Psychiatrica Scandinavica. (2012) 125:440–52. 10.1111/j.1600-0447.2012.01826.x 22242976

[16] Zhang G Guo Y Peng X Dong F Song J. Impact of perspective-taking and empathy on HIV-related stigma among college students in Guilin. Chin J School Health. (2020) 41:1319–21.

[17] Fangen K Lynnebakke B. Navigating ethnic stigmatisation in the educational setting: Coping strategies of young immigrants and descendants of immigrants in Norway. Soc Inclusion. (2014) 2:47–59. 10.17645/si.v2i1.26

[18] McKenzie K. Stigma, race, and culture. Psychiatr Serv. (2014) 65:963–63. 10.1176/appi.ps.650603 26037010

[19] Decker SN Ortiz N Spohn C Hedberg E. Criminal stigma, race, and ethnicity: The consequences of imprisonment for employment. J Crim Justice. (2015) 43:108–21. 10.1016/j.jcrimjus.2015.02.002

[20] Cokley K Awad G Smith L Jackson S Awosogba O Hurst A. (2015). The roles of gender stigma consciousness, impostor phenomenon and academic self-concept in the academic outcomes of women and men. Sex Roles. (2015) 73:414–26. 10.1007/s11199-015-0516-7

[21] Lacey M Paolini S Hanlon MC Melville J Campbell LE. Parents with serious mental illness: Differences in internalised and externalised mental illness stigma and gender stigma between mothers and fathers. Psychiatry Res. (2014) 225:723–33. 10.1016/j.psychres.2014.09.010 25524813

[22] Wu CL. Stigmatized gender and gendered stigma: the “infertile” men and women in Taiwan. Taiwanese J Sociol. (2002) 29:127–79.

[23] Chen Y Li H. Influence of gender stereotypes on the attribution of success and failure of males and females. J Hubei Univ Sci Technol. (2014) 34:156–7. 10.3969/j.issn.1006-5342.2014.05.074

[24] Fu J. Gender justice and educational recognition. J Yunnan Minzu University (Social Sciences). (2014) 31:72–5.

[25] Lian S. Improving the chances of female undergraduates in employment: from the perspective of implicit gender stereotypes. Ideol Theor Edu. (2014) 20:87–91.

[26] Zhu X Liao Q IAT IAT based research on implicit occupational gender stereotypes. Adv Psychol. (2020) 10:124–8. 10.12677/AP.2020.102016

[27] Bayat AH Mohammadi R Moradi-Joo M Bayani A Ahounbar E Higgs P. HIV and drug related stigma and risk-taking behaviors among people who inject drugs: a systematic review and meta-analysis. J Addict Dis. (2020) 38:1–13. 10.1080/10550887.2020.1718264 32186479

[28] Calabrese SK Burke SE Dovidio JF Levina OS Uusküla A Niccolai LM. Internalized HIV and drug stigmas: Interacting forces threatening health status and health service utilization among people with HIV who inject drugs in St. Petersburg. Russia AIDS Behav. (2016) 20:85–97. 10.1007/s10461-015-1100-4 26050155 PMC4793904

[29] Morrison TG Whitehead BW. “Nobody's ever going to make a fag pretty woman: Stigma awareness and the putative effects of stigma among a sample of Canadian male sex workers. J Homosex. (2008) 53:201–7. 10.1300/J082v53n01_09 18019075

[30] Nardelli N Baiocco R Tanzilli A Lingiardi V. Not in the same mental drawer: Internalized sexual stigma, dissociation, and the role of religion in a sample of Italian gay men. J Homosex. (2020) 67:1386–400. 10.1080/00918369.2019.1591786 30912733

[31] Salvati M Pellegrini V Giacomantonio M Cristofaro VD. Embrace the leadership challenge: The role of gay men's internalized sexual stigma on the evaluation of others' leadership and one's own. Br J Soc Psychol. (2020) 60:700–19. 10.1111/bjso.12424 33044021

[32] Chen X Bi L Yu Y. University students' implicit identity-related stigma towards migrant workers. Chin J Clin Psychol. (2013) 21:372–5.

[33] Li X Li J. Stigmatization and double constructions of peasant-workers' subjectivity. J Shanxi Agric Univ. (2018) 17:29–35. 10.13842/j.cnki.issn1671-816x.2018.06.005

[34] Xu J Jiang L. Impact of social stigma and discrimination on the mental health of the new-generation migrant workers: an analysis of the mediating role of several factors. Chin J Health Policy. (2018) 11:52–61. j.issn.1674-2982.2018.06.009

[35] Zhao D. Effects of stigmatized identities on interpersonal influence and social distance. Acta Psychologica Sinica. (2013) 45:1283–94. 10.3724/SP.J.1041.2013.01283

[36] Zhang Y. The stigmatic situation and the coping strategies: a case study of the adaptation of the floating population to urban life and the community change. Society. (2008) 28:126–47.

[37] Song X. Causes for violent law enforcement in urban management based on stigmatization theories and countermeasures. Modern Bus Trade Ind. (2020) 30:158–9. 10.19311/j.cnki.1672-3198.2020.08.074

[38] Cheng M. Cultural cost of self-made man: a traveler to the upper class. China Youth Study. (2016) 28:91–7. 10.3969/j.issn.1002-9931.2016.12.014

[39] Wang W. Study of the stigmatization of self-made man from the perspective of sociology. China Youth Study. (2014) 26:73–9. 10.5005/jp/books/12160_7

[40] Xu X. A study of the marriage narrative, identity production and stigmatization of the “phoenix man”. Hum Soc Sci J Hainan Univ. (2020) 38:93–8.

[41] Lei L Feng D Tan X. Development and preliminary application of adolescents online stigmatization. Chin J Clin Psychol. (2012) 20:328–31. 10.16128/j.cnki.1005-3611.2012.03.015

[42] Ritsher JB Otilingam PG Grajales M. Internalized stigma of mental illness: psychometric properties of a new measure. Psychiatry Res. (2003) 121:31–49. 10.1016/j.psychres.2003.08.008 14572622

[43] Tan X Feng D Lei L. Relationship between Teenagers' Stigma of Internet Surfing and 'Internet Addiction. In: *Proceedings of the 15th National Academic Congress of Psychology* Guangzhou (2012).

[44] Ugrin JC Pearson JM. The effects of sanctions and stigmas on cyberloafing. Comput Human Behav. (2013) 29:812–20. 10.1016/j.chb.2012.11.005

[45] Peter SC Li Q Pfund RA Whelan JP Meyers AW. Public stigma across addictive behaviors: Casino gambling, esports gambling, and internet gaming. J Gambl Stud. (2018) 35:247–59. 10.1007/s10899-018-9775-x 29627881

[46] Jiang Q Tong S Chen Z. Teenagers' dependence on the internet and risk response. Youth Explor. (2020) 38:37–46. 10.13583/j.cnki.issn1004-3780.2020.06.004

[47] China Internet Network Information Center, CNNIC. 2020 China National Research Report on Internet Usage for Minors [OB/OL] (2021). Available online at: http://www.cnnic.net.cn/hlwfzyj/hlwxzbg/qsnbg/202107/P020210720571098696248.pdf (accessed July 20, 2021).

[48] Ji W. Trend in teenagers' use of the internet and its influence on their growth in the era of social media: an analysis of the survey on teenagers' use of the internet. News and Writing. (2020) 37:43–50.

[49] Li Y. (2020). Characteristics of the internet behavior of undergraduates in the new era and their pattern of change. Young Soc. (2020) 14:151–2.

[50] Peng S Zhang XY Zhang HP Hu XG. The relationship between fear of negative evaluation and internet overuse: the mediating effects of social anxiety and self-control. J Psychol Sci. (2020) 43:81–6. 10.16719/j.cnki.1671-6981.20200112

[51] Cui Y Yang YT Qian H Cui W Cui LJ. Analysis on the related factors of college students' network use and internet addiction. Med Res Edu. (2020) 3:55–61. 10.3969/j.issn.1674-490X.2020.05.00833762997

[52] Johnson S E Lawrence D Hafekost J Saw S Buckingham WJ Sawyer M. Service use by Australian children for emotional and behavioural problems: Findings from the second Australian child and adolescent survey of mental health and wellbeing. Aust N Z J Psychiatr. (2016) 50:887–98. 10.1177/0004867415622562 26769979

[53] Posso A. Internet usage and educational outcomes among 15-year-old Australian students. Int J Commun. (2016) 10:3851–76.

[54] Thomas NJ Martin FH. Video-arcade game, computer game and internet activities of Australian students: participation habits and prevalence of addiction. Aust J Psychol. (2011) 62:59–66. 10.1080/00049530902748283

[55] Rahamathulla M Rohde A Rajik N Quinn S. Problematic internet use and co-morbid mental health problems among university students in Australia. Int J Technol Knowl Soc. (2020) 16:1–11. 10.18848/1832-3669/CGP/v16i01/1-11

[56] Rikkers W Lawrence D Hafekost J Zubrick SR. Internet use and electronic gaming by children and adolescents with emotional and behavioural problems in Australia - results from the second child and adolescent survey of mental health and wellbeing. BMC Public Health. (2016) 16:399. 10.1186/s12889-016-3058-1 27178325 PMC4866411

[57] King DL Delfabbro PH DohYY Wu AMS. Sakuma H. Policy and prevention approaches for disordered and hazardous gaming and internet use: an international perspective prevention. Science. (2018) 19:233–49. 10.1007/s11121-017-0813-1 28677089

[58] Leung C Moore SM Karnilowicz W Lung CL. Romantic relationships, relationship styles, coping strategies, and psychological distress among Chinese and Australian young adults. Soc Dev. (2011) 20:783–804. 10.1111/j.1467-9507.2011.00616.x

[59] Hu J Wang Z. Exploring the associated factors of elevated psychological distress in a community residing sample of Australian Chinese migrants. Aust J Psychol. (2016) 68:116–22. 10.1111/ajpy.12099

[60] Byrne BM Shavelson RJ Muthén BO. Testing for the equivalence of factor covariance and mean structures: the issue of partial measurement invariance. Psychol Bull. (1989) 105:456–66. 10.1037/0033-2909.105.3.456

[61] Schmitt N Kuljanin G. Measurement invariance: review of practice and implications. Hum Resour Manag Rev. (2008) 18:210–22. 10.1016/j.hrmr.2008.03.00317892098

[62] Vandenberg RJ Lance CE. A. review and synthesis of the measurement invariance literature: Suggestions, practices, and recommendations for organizational research. Organ Res Methods. (2000) 3:4–69. 10.1177/109442810031002

[63] Chen FF. What happens if we compare chopsticks with forks? The impact of making inappropriate comparisons in cross-cultural research. J Pers Soc Psychol. (2008) 95:1005–18. 10.1037/a0013193 18954190

[64] Byrne BM Watkins D. The issue of measurement invariance revisited. J Cross Cult Psychol. (2003) 34:155–75. 10.1177/0022022102250225

[65] Raju NS Laffitte LJ Byrne BM. Measurement equivalence: a comparison of methods based on confirmatory factor analysis and item response theory. J Appl Psychol. (2002) 87:517–29. 10.1037/0021-9010.87.3.517 12090609

[66] Reise SP Widaman KF Pugh RH. Confirmatory factor analysis and item response theory: Two approaches for exploring measurement invariance. Psychol Bull. (1993) 114:552–66. 10.1037/0033-2909.114.3.552 8272470

[67] Hu L Bentler PM. Cutoff criteria for fit indexes in covariance structure analysis: Conventional criteria versus new alternatives. Struct Equ Model. (1999) 6:1–55. 10.1080/10705519909540118

[68] Chen FF. Sensitivity of goodness of fit indexes to lack of measurement invariance. Struct Equ Model. (2007) 14:464–504. 10.1080/10705510701301834

[69] Cheung GW Rensvold RB. Evaluating goodness-of-fit indexes for testing measurement invariance. Struct Equ Model. (2002) 9:233–55. 10.1207/S15328007SEM0902_522551991

[70] Tavakol M Dennick R. Making sense of Cronbach's alpha. Int J Med Edu. (2011) 2:53–5. 10.5116/ijme.4dfb.8dfd 28029643 PMC4205511

[71] Clark L A Watson D. Constructing validity: basic issues in objective scale development. Psychol Assess. (1995) 7:309–19. 10.1037/1040-3590.7.3.309

[72] Bieda A Hirschfeld G Schönfeld P Brailovskaia J Zhang X C Margraf J. Universal happiness? Cross-cultural measurement invariance of scales assessing positive mental health. Psychol Assess. (2017) 29:408–21. 10.1037/pas0000353 27322203

[73] Whisman MA Judd CM. A cross-national analysis of measurement invariance of the satisfaction with life scale. Psychol Assess. (2016) 28:239–44. 10.1037/pas0000181 26168309

[74] Barnette JJ. Effects of stem and Likert response option reversals on survey internal consistency: if you feel the need, there is a better alternative to using those negatively worded stems. Educ Psychol Meas. (2000) 60:361–70. 10.1177/00131640021970592

[75] Wong N Rindfleisch A Burroughs JE. Do reverse-worded items confound measures in cross-cultural consumer research? The case of the material values scale. J Consum Res. (2003) 30:72–91. 10.1086/374697

[76] Marsh HW Scalas LF Nagengast B. Longitudinal tests of competing factor structures for the Rosenberg Self-Esteem Scale: traits, ephemeral artifacts, and stable response styles. Psychol Assess. (2010) 22:366–81. 10.1037/a0019225 20528064

[77] Weijters B Baumgartner H Schillewaert N. Reversed item bias: an integrative model. Psychol Methods. (2013) 18:320–34. 10.1037/a0032121 23646990

[78] Wu Y Wei QW Zhou ZM. Culture and social psychology. Intellectual Property Publishing House Co, Ltd Beijing (2017).

[79] Smith PB Fischer R Vignoles VL Bond MH. Understanding social psychology across cultures (2nd Ed). London: Sage Publications Ltd (2013).

[80] King D L Russell A Hing N. Adolescent land-based and internet gambling: Australian and international prevalence rates and measurement issues. Curr Addic Rep. (2020) 7:137–48. 10.1007/s40429-020-00311-1

[81] Hutton V Sisko S. Multicultural Responsiveness in Counselling and Psychology: Working with Australian Populations. Cham: Palgrave Macmillan Cham. (2021).

[82] Morgan AJ Wright J Reavley NJ. Review of Australian initiatives to reduce stigma towards people with complex mental illness: What exists and what works? Int J Ment Health Syst. (2021)15:10. 10.1186/s13033-020-00423-1 33461567 PMC7814561

[83] SANE Australia. A life without stigma: A SANE Report. SANE Australia (2013)

[84] Wright A Jorm AF Mackinnon AJ. Labeling of mental disorders and stigma in young people. Soc Sci Med. (2011) 73:498–506. 10.1016/j.socscimed.2011.06.015 21794967

[85] Sheikhmiri M Issa T. Awareness factors, opportunities and challenges of iot application in Australia. In: Issa T, Issa T, Issa TB, Isaias P, editors. Sustainability awareness and green information technologies. Green Energy and Technology. Cham: Springer (2020) 271–320. 10.1007/978-3-030-47975-6_12

